# Inflammation-modulating agents in chronic limb-threatening ischemia: a narrative review of therapeutic potential and future directions

**DOI:** 10.1097/MS9.0000000000004053

**Published:** 2025-10-28

**Authors:** Aditya Gaur, Precious Dike, Timilehin Mayowa, Favour. C. Mekowulu, Anas Abdulkader, Medha Sridhar Rao, Awogboro Shola, Promise Ajala, Oderinde Oluwatobi, Israel Charles Abraham, Emmanuel Kokori, Gbolahan Olatunji, Nicholas Aderinto

**Affiliations:** a Department of Medicine, Yeovil District Hospital, Somerset NHS Foundation Trust, Higher Kingston, Yeovil, United Kingdom; b Department of Medicine, Watford General Hospital, West Hertfordshire Teaching Hospitals Trust, Watford, United Kingdom; c Department of Medicine, University College Hospital, Ibadan, Nigeria; d Department of Medicine, Specialist Practice for Cardiology and Pulmonology Eggenfelden, Germany; e College of Medicine, AlMaarefa University, Riyadh, Saudi Arabia; f School of Medicine, Dentistry and Biomedical Sciences, Queen’s University Belfast, Belfast, United Kingdom; g Department of Medicine, Obafemi Awolowo University, Ife, Nigeria; h College of Medicine, University of Ibadan, Ibadan, Nigeria; i Department of Medicine and Surgery, University of Ilorin, Ilorin, Nigeria; j Department of Medicine and Surgery, Ladoke Akintola University of Technology, Ogbomoso, Nigeria

**Keywords:** canakinumab, chronic limb-threatening ischemia, colchicine, inflammation, NLRP3 inflammasome

## Abstract

Chronic limb-threatening ischemia (CLTI) represents the most severe form of peripheral artery disease (PAD), driven by both advanced atherosclerosis and chronic inflammation. Despite advancements in revascularization and medical therapy, outcomes remain poor. Inflammation has emerged as a key therapeutic target, and several anti-inflammatory agents are under investigation for potential benefit in CLTI. A comprehensive literature review was conducted using PubMed, Scopus, Embase, Google Scholar, and Web of Science to identify studies published between 1990 and 2025. Keywords included “chronic limb-threatening ischemia,” “peripheral artery disease,” “colchicine,” “canakinumab,” “methotrexate,” “NLRP3 inflammasome,” and “anti-inflammatory therapy.” Eligible studies included original research, clinical trials, systematic reviews, and relevant guidelines focused on inflammation-targeted interventions in atherosclerosis and limb ischemia. Colchicine has shown significant reductions in major adverse cardiovascular events in large trials and retrospective PAD studies, though no randomized controlled trials (RCTs) have specifically addressed CLTI. Canakinumab reduced inflammatory biomarkers and improved walking performance in PAD but had no effect on plaque progression. Methotrexate did not demonstrate cardiovascular benefit in the Cardiovascular Inflammation Reduction Trial. Preclinical agents targeting the NLRP3 inflammasome (e.g., MCC950) have shown anti-inflammatory effects but lack human data. Nutraceuticals, including omega-3 fatty acids and polyphenols, may offer adjunctive benefits, though evidence remains limited. Inflammation-modulating therapies hold promise as adjuncts in CLTI management, potentially improving outcomes by targeting the underlying immune mechanisms of disease progression. However, current evidence is insufficient for clinical adoption in CLTI. Large-scale, well-designed RCTs focusing on limb-specific outcomes are necessary to clarify the role of these therapies in high-risk vascular populations.

## Introduction

Chronic limb-threatening ischemia (CLTI), also popularly referred to as critical limb ischemia in some contexts, is an arterial disorder marked by symptomatic reduction in blood flow to the limbs. Although most commonly caused by atherosclerosis and, less frequently, vasculitis, CLTI primarily affects the lower extremities, with occasional involvement of the upper limbs and gluteal region. CLTI represents the most severe clinical form of peripheral arterial disease (PAD) and is defined by ischemic rest pain, non-healing ulcers, or gangrene persisting for more than 2 weeks, in conjunction with objectively verified arterial occlusive disease^[1]^. This diagnosis signifies a state of severely impaired perfusion, leading to tissue hypoxia and cellular dysfunction. Affecting up to 1% of individuals over age 50, the prevalence of CLTI is expected to rise due to increasing rates of diabetes mellitus and atherosclerosis worldwide^[2]^.

The clinical burden of CLTI is profound. Without timely and effective treatment, major limb amputation may become necessary, with approximately 25% of patients undergoing amputation within 1 year of diagnosis^[3]^. Even following successful revascularization, healing complications persist, as up to 50% of patients experience recurrent or non-healing ulceration at 1 year^[4]^. Moreover, CLTI is a strong marker of systemic atherosclerosis and is associated with high mortality, with over 20% of patients dying within 1 year of diagnosis. Five-year survival rates are comparable to those seen in other chronic diseases^[5]^. While limb ischemia is often viewed as the principal cause of mortality, many patients ultimately succumb to associated major adverse cardiovascular and cerebrovascular events, indicative of widespread vascular involvement^[6]^.

While traditionally understood as a result of arterial occlusion from atherosclerosis and thrombosis, CLTI is now recognized as a complex condition in which chronic inflammation plays a central role. Inflammatory cells such as macrophages and neutrophils infiltrate ischemic tissue and release cytokines, proteases, and reactive oxygen species (ROS) that can worsen injury rather than promote repair^[7]^. Elevated levels of systemic inflammatory markers, including C-reactive protein (CRP), interleukin-6 (IL-6), and tumor necrosis factor-alpha (TNF-α), are associated with poorer outcomes, including an increased risk of amputation and death^[8]^. Despite growing evidence, current clinical guidelines do not recommend anti-inflammatory therapies for CLTI^[9,10]^, with existing treatments focusing on restoring perfusion and managing metabolic contributors through antiplatelet and lipid-lowering therapy, glycemic control, and wound care^[11]^. This review aims to assess the role of inflammation in CLTI pathophysiology and evaluate existing evidence supporting anti-inflammatory interventions, with emphasis on inflammatory mediators such as the NLRP3 inflammasome and cytokines like interleukin-1β (IL-1β) and IL-6^[12]^. It will also explore anti-inflammatory strategies used in related conditions to identify potential translational approaches for improving outcomes in CLTI. This paper adheres to the Transparency In The reporting of Artificial INtelligence (TITAN) guidelines^[13]^.

## Methods

A comprehensive literature search was conducted using PubMed, Scopus, Embase, Google Scholar, and Web of Science to identify relevant studies related to inflammation, CLTI, and anti-inflammatory therapies in PAD. The search was limited to articles published between 1996 and 2025 to ensure inclusion of both foundational and up-to-date evidence. Search terms and Boolean combinations included “chronic limb-threatening ischemia,” “peripheral artery disease,” “atherosclerosis,” “vascular inflammation,” “NLRP3 inflammasome,” “IL-1β,” “IL-6,” “colchicine,” “canakinumab,” “methotrexate,” “tocilizumab,” “omega-3 fatty acids,” “polyphenols,” and “anti-inflammatory therapy.”

Inclusion criteria were original research articles, clinical trials, systematic reviews, meta-analyses, and relevant clinical guidelines discussing inflammation in PAD or CLTI, with emphasis on cytokine signaling, inflammatory markers, molecular pathways, and investigational or established anti-inflammatory interventions. Studies based on animal models, non-atherosclerotic etiologies of limb ischemia, and pediatric populations were excluded.

Reference lists of key publications were screened to identify additional sources. Extracted data were synthesized into a narrative framework exploring the pathophysiological role of inflammation in CLTI and the therapeutic potential of anti-inflammatory agents, including both approved treatments and investigational compounds.

**HIGHLIGHTS**

- Chronic limb-threatening ischemia (CLTI) is driven by atherosclerosis and inflammation, with biomarkers like C-reactive protein, interleukin-6, and tumor necrosis factor-alpha linked to worse outcomes.
- Colchicine reduces cardiovascular and limb events in peripheral artery disease, and canakinumab improves walking performance but not plaque progression, while methotrexate shows no benefit in atherosclerosis.
- Lack of CLTI-specific trials limits the adoption of anti-inflammatory therapy.

## The inflammatory nature of CLTI

### Pathophysiology

CLTI represents a distinct clinical progression of PAD, marked by persistent ischemic symptoms and objective arterial obstruction^[14]^. While its association with advanced atherosclerosis is well established, emerging evidence emphasizes the role of chronic inflammation as a core element in its development and persistence^[15]^. PAD itself is frequently accompanied by elevated levels of systemic inflammatory markers, and these biomarkers have been valuable in assessing both disease progression and patient prognosis^[16,17]^. Importantly, the vascular changes in CLTI are not solely mechanical; rather, the surrounding tissue environment is significantly shaped by inflammatory signaling and immune cell activity, which complicate the ischemic process^[18]^.

Atherosclerotic progression begins with endothelial dysfunction – often triggered by cardiovascular risk factors such as smoking, diabetes, and hyperlipidemia – and quickly evolves into an inflammatory cascade involving monocyte infiltration and cytokine release^[16,19]^. This leads to the production of molecules such as IL-1β, IL-6, CRP, MCP-1, Matrix metalloproteinase (MMPs), and a range of adhesion proteins^[20,21]^. These mediators collectively foster plaque instability and ongoing immune activation within the vascular wall. The response is further sustained by extracellular vesicles and adhesion molecules that maintain leukocyte recruitment and vascular injury. In this context, inflammation is not a secondary feature but a continuous contributor to the structural and functional decline of the arterial system.

As vessels become increasingly compromised, the resulting hypoxia sets off a chain reaction of maladaptive responses – ranging from microvascular thrombosis and platelet aggregation to tissue edema and cellular stress^[21]^. Although angiogenic mechanisms are triggered in an attempt to restore perfusion, these efforts are often insufficient or counterproductive, occasionally leading to destabilization of upstream plaques^[18]^. Moreover, repeated ischemic episodes provoke systemic inflammatory surges, compounding local tissue injury and hindering regenerative efforts^[16]^. The resulting microenvironment is characterized by impaired wound repair and persistent inflammation, which together sustain the pathological cycle seen in CLTI^[14]^.

### Key inflammatory biomarkers

Multiple studies have highlighted a strong connection between specific inflammatory markers and the clinical behavior of CLTI, including its onset, severity, and treatment outcomes. Elevated levels of pro-inflammatory markers such as TNF-α, IL-6, CRP, the neutrophil-to-lymphocyte ratio (NLR), and fibrinogen have been observed in patients with CLTI, offering insight into the underlying inflammatory activity^[17,19]^. These markers, while varied in their biological roles, collectively reflect a systemic immune imbalance that contributes to ongoing tissue damage and impaired recovery.

TNF-α, a pleiotropic cytokine, plays an essential role in cellular processes like inflammation, cell survival, and angiogenesis. Its increased expression in CLTI patients – particularly those with diabetes – has been linked to worsening ischemia and poor vascular outcomes^[19,20]^. IL-6, known for its function in stimulating antibody production and T-cell responses, is similarly elevated and associated with a higher likelihood of adverse events, including limb loss or major cardiovascular complications post-revascularization^[19]^. The NLR, a readily accessible marker of systemic inflammation, has proven useful in predicting disease progression, limb amputation, and long-term cardiovascular mortality^[17,20]^. CRP, long established as a marker of acute inflammation, not only reflects ongoing vascular injury but also plays an active role in atheroma development and has been linked to a higher risk of myocardial infarction, stroke, and adverse limb events^[17,19]^.

**Figure 1.**
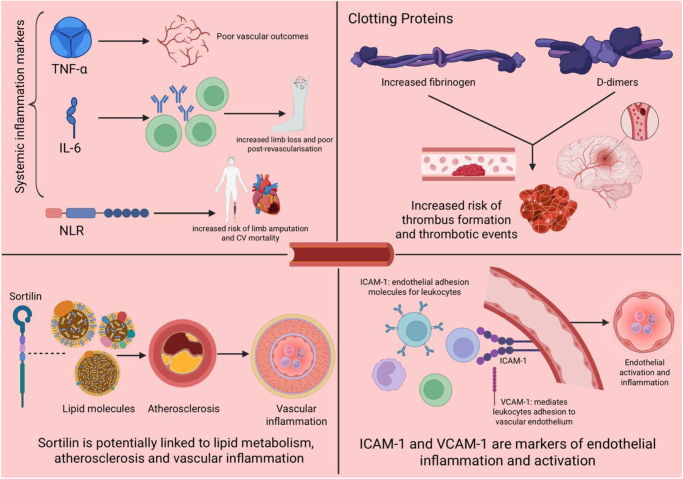
Pathophysiologic model of ischemia–inflammation–tissue loss in chronic limb-threatening ischemia.

Fibrinogen, another acute-phase reactant, contributes to clot formation and increased blood viscosity. Elevated fibrinogen levels in CLTI have been tied to greater risks of thrombotic complications, including graft occlusion and stroke, and are considered a poor prognostic indicator, particularly among elderly patients^[22,23]^. Beyond these, additional biomarkers – such as D-dimers, ICAM-1, VCAM-1, E-selectin (CD-62), HMGB-1, neopterin, osteoprotegerin (OPG), and sortilin – have also been studied for their potential roles in vascular inflammation and endothelial dysfunction in this patient population^[19]^. These emerging indicators not only underscore the systemic inflammatory burden of CLTI but also may offer opportunities for future diagnostic refinement and targeted therapy. A comprehensive summary of the role of the biomarkers involved in CLTI has been listed in Figure 1 below.

### Tissue-level and molecular evidence

There is much evidence at the microscopic level that lends credence to the theory that inflammation is a driving force in CLTI.

#### Neutrophil/macrophage infiltration

Aside from their pivotal role in innate immunity, studies also indicate the importance of neutrophils in the modulation of the adaptive immune response. Neutrophils are polymorphonuclear and phagocytic leukocytes involved in immune regulation during both the innate and adaptive immune responses and are, therefore, considered as therapeutic targets in several diseases, such as atherosclerosis^[24]^. They comprise the first line of host defense in response to invading pathogens, where chemotaxis, phagocytosis, and the release of ROS and cytokines occur by these cells^[19]^. Their activation is found to be notably higher in the ischemic limb^[16]^. Once activated, they release proteases and ROS, contributing further to tissue damage^[17]^.

Monocytes and macrophages are examples of other immune cells that are known to enhance this immune response by generating interleukin-8 (IL-8), which has the function of attracting additional neutrophils and monocytes to the site of inflammation^[19]^.

#### Elevated cytokine signaling

NLR-dependent recognition of external or internal danger signals triggers the recruitment of adaptor proteins and the formation of molecular platforms known as inflammasomes. The NLRP3 inflammasome constitutes a high molecular complex that regulates cytokine maturation and is implicated, directly or indirectly, in the pathogenesis of a wide range of disorders^[25]^.

NLRP3 contributes to systemic inflammatory response in patients with PAD, due to its role in oxidative stress and inflammatory vascular environments, giving further rationale to the need to address PAD with anti-inflammatory agents. As more and more studies lend weight to the crucial role of inflammation in PAD, clinical trials have begun to investigate the efficacy of therapeutic inhibition of subsequent downstream inflammasome markers, specifically IL-1β and caspase-1, in pathologies associated with PAD and calcification such as type II diabetes and atherosclerosis^[26]^.

## Anti-inflammatory mechanisms of interest

### Cytokine-targeted pathways

It has been known for quite some time that the immune system plays a big part in how atherosclerosis develops^[27]^. When oxidized low-density lipoprotein (LDL) and other modified lipids build up inside artery walls, they trigger an immune response that damages the endothelium. This draws in mononuclear cells, which turn into macrophages and form foam cells inside plaques. These foam cells produce inflammatory cytokines like IL-1β, which drive inflammation and create necrotic areas within the plaques^[28]^. IL-1β contributes to the disease in several ways – it promotes blood clotting activity, helps white blood cells stick to vessel walls, and encourages smooth muscle cells in the vessel to grow^[29]^. Macrophages activated by IL-1α release an inactive IL-1β precursor that becomes active through cleavage by enzymes inside the cell or from neutrophils outside the cell. This process also increases VCAM1 expression, which helps more monocytes move in and produces even more IL-1β. Drugs like canakinumab specifically block IL-1β and show the potential for targeted treatment^[30]^.

Blocking IL-1β looks promising for slowing down atherosclerosis because it plays a big role in many parts of the disease process. The NLRP3 inflammasome is a key activator of IL-1β, responding to things like cholesterol crystals, neutrophil traps, low oxygen levels, and blood flow disturbances – all important drivers of plaque formation^[8]^. Genetic studies have linked IL-1β activity to IL-6 receptor pathways, which contribute to clot formation in arteries. New research using mouse models and studying blood cell mutations has also tied IL-1β to faster atherosclerosis via bone marrow activation^[31]^. In older adults, genes controlling inflammasome activity and IL-1β are linked to higher death rates and worse atherosclerosis^[32]^. The Canakinumab Anti-inflammatory Thrombosis Outcomes Study (CANTOS) trial provided strong clinical evidence that inhibiting IL-1β could reduce cardiovascular events, marking a major step forward in treating this disease^[33]^.

IL-6 is another important cytokine, especially when it comes to inflammation in chronic wounds and ischemic tissue damage. It helps with immune cell development and stimulates the production of antibodies and T cells^[34]^. IL-6 levels rise quickly after injury or infection but drop once the problem resolves, thanks to mechanisms that degrade its mRNA^[19]^. Elevated IL-6 is seen in vascular diseases and is linked with worse outcomes in diabetic patients with critical limb ischemia after revascularization, including higher risks of amputations and serious cardiovascular problems^[35]^.

While IL-1β and IL-6 are well studied in heart disease, the inflammatory signals in PAD might differ. For example, in PAD, especially milder cases like claudication, TNF-α and IL-8 often show higher levels than IL-6. Still, studies generally find increased IL-6, TNF-α, selectins, neopterin, adhesion molecules, and matrix metalloproteinases in PAD patients^[36]^. However, IL-6 levels can be inconsistent, and in some cases, they do not differ much from IL-1β in lower limb ischemia^[37]^. The CANTOS trial was important because it showed that targeting IL-1β and IL-6 pathways can reduce cardiovascular risk independent of traditional risk factors, highlighting new possibilities for treatment in ischemic diseases^[8]^.

### NLRP3 inflammasome

The activation of the NLRP3 inflammasome is gaining attention as a key player in vascular inflammation and may represent a promising target for treating PAD. Bartoli-Leonard *et al* (2023)^[26]^ found that NLRP3 levels are higher in arteries affected by PAD, especially in areas rich in macrophages, and its presence correlates with vascular calcification and widespread inflammation. Specifically, NLRP3 was elevated in both the intima and media layers of diseased arteries compared to healthy controls, often coexisting with CD68-positive macrophages in the intima and smooth muscle actin in the media^[38]^. This expression also showed a positive association with the extent of calcification and circulating CRP levels^[26]^.

In a different study, Vogel *et al* (2019)^[39]^ explored the role of platelet NLRP3 inflammasome activation in hindlimb ischemia using a mouse model where the femoral artery was ligated. They observed increased inflammasome activity in circulating platelets after the artery was tied off, demonstrated by higher caspase-1 activation and IL-1β cleavage. Importantly, mice that either lacked TLR4 in their platelets or had defective NLRP3 showed better blood flow recovery in the affected limb compared to normal mice. This suggests that the platelet NLRP3 inflammasome contributes to impaired reperfusion after ischemic injury^[39]^.

NLRP3 acts as a crucial mediator of vascular and wound inflammation by activating IL-1β, a process enhanced by factors such as tissue hypoxia, fatty deposits, and disturbed blood flow, all of which promote atherosclerosis progression^[33]^. Bartoli-Leonard *et al* (2023)^[26]^ further examined arteries from PAD patients and found significantly higher levels of pro-inflammatory cytokines, including IL-1β, TNF-α, and IL-33, compared to controls, confirming NLRP3 activation. Their results linked NLRP3 activation to macrophage buildup and calcification in PAD arteries, highlighting its possible role in disease progression^[26]^. Because inhibiting NLRP3 can reduce both IL-1 and interleukin-18 (IL-18) production, it holds therapeutic potential, especially since IL-18 may mediate some heart dysfunction caused by IL-1^[40]^. Platelets mainly use two pattern recognition receptors – TLR4 and NLRP3 – and TLR4 activation can trigger NLRP3 inflammasome formation. This interaction increases platelet aggregation and clot formation, influencing limb blood flow and muscle damage after ischemic injury. Vogel *et al* (2019)^[39]^ suggested that in their mouse model, TLR4-driven NLRP3 activation in platelets plays a key role in regulating these effects after hindlimb ischemia.

### Neutrophil and macrophage activation

PAD is now widely recognized as a chronic inflammatory condition, where inflammation plays a major role in how the disease develops and worsens over time. The buildup of atherosclerotic plaques inside the arteries triggers an immune response, recruiting cells like neutrophils and macrophages. This inflammation damages the lining of blood vessels, makes plaques unstable, narrows arteries, and contributes to the progression of PAD and the risk of developing CLTI^[41]^.

Atherosclerosis develops slowly, driven by problems like endothelial dysfunction, oxidative stress, inflammation, and high LDL cholesterol, which cause fat to build up inside artery walls^[42]^. Monocytes move into the tissue beneath the endothelium and turn into macrophages. Depending on their surroundings, these macrophages can take on different roles. They absorb LDL particles, becoming foam cells that fuel plaque growth by releasing inflammatory signals and breaking down tissue structures. This ongoing process weakens plaques, making them more likely to rupture^[43]^. Macrophages are quite adaptable during muscle repair; they shift between different forms. Initially, neutrophils worsen injury, then inflammatory M1 macrophages clear dead cells and promote inflammation. Later on, M2 macrophages take over, producing anti-inflammatory factors and encouraging tissue healing by supporting extracellular matrix buildup. M2 cells also help recruit stem cells after injury. Higher levels of M1 macrophages have been linked with poorer walking function, while M2 macrophages aid the growth of new blood vessels^[44]^.

Studies suggest that the variation in inflammatory responses between patients with CLTI and those with claudication may reflect the severity and activity of the underlying atherosclerosis^[22]^. In individuals with critical limb ischemia, there tends to be a higher presence of pro-inflammatory M1 macrophages compared to the anti-inflammatory M2 subtype. M1 macrophages release cytokines such as IL-1β, IL-6, and interleukin-12, which drive inflammation, whereas M2 macrophages produce anti-inflammatory cytokines like interleukin-4 and interleukin-13 that promote healing^[45]^. Unstable, progressive plaques are generally dominated by M1 macrophages, while more stable plaques contain a greater number of M2 macrophages that encourage fibrotic tissue repair. This imbalance in macrophage activity within plaques likely contributes to the worsening of disease, helping explain why some patients transition from intermittent claudication to the more severe CLTI^[45]^.

## Existing therapies with anti-inflammatory effects

### Statins

Statins are essential in treating PAD because they reduce cardiovascular risks and improve limb-related outcomes. Although current guidelines recommend statins for all PAD patients to lower the risk of complications and death, they are prescribed far less often than in coronary heart disease cases^[46]^. High-intensity statins, which offer the greatest benefit, remain underutilized, with only around 36% of eligible patients receiving them^[47]^. Statin use is linked to a roughly 30% drop in major adverse limb events (MALE), a 35% decrease in amputation rates, 39% lower overall mortality, and 41% less cardiovascular mortality^[48]^. The benefit is particularly notable in patients with high CRP levels, where statins’ anti-inflammatory effects translate into better limb salvage and reduced mortality^[49]^. Additionally, statins protect kidney function following angiography or interventions^[50]^, and compared to lower doses, high-intensity regimens significantly improve survival and cut major cardiovascular events^[51]^. Statins also help maintain artery patency after lower-limb angioplasty, reducing limb loss and death^[52]^, and support graft durability by lowering restenosis and amputation after infrainguinal bypass surgery^[53]^. Despite these benefits, patients with widespread vascular disease continue to face high mortality even on optimal statin therapy, suggesting the need for stronger lipid-lowering approaches^[54]^. Poor adherence – especially among older women due to higher copayments and decreased quality of life – is an ongoing challenge^[55]^. Overall, statins remain a cornerstone of PAD treatment, but better compliance is essential.

Statins’ effects extend beyond lowering cholesterol. They have a strong anti-inflammatory impact independent of lipid changes, consistently reducing CRP levels by 40–50%, regardless of LDL response^[56]^. This anti-inflammatory action is crucial in PAD and CLTI, where inflammation drives disease progression. Patients with elevated CRP levels see the greatest benefits, including improved limb salvage and fewer cardiovascular events^[49,57]^. Statins also boost the availability of nitric oxide (NO) by increasing endothelial nitric oxide synthase (eNOS) activity, which helps blood vessels relax. For instance, simvastatin and atorvastatin raise eNOS expression and NO production by about 25–30%, supporting vascular health and lowering neutrophil adhesion to vessel walls^[58,59]^. Additionally, they influence L-arginine metabolism, further encouraging NO synthesis^[59]^.

Moreover, statins reduce oxidative stress by decreasing nicotinamide adenine dinucleotide phosphate (NADPH) oxidase activity and enhancing eNOS function, which cuts down the formation of ROS and protects the vascular endothelium^[60,61]^. This results in fewer adhesion molecules that attract inflammatory cells, limiting plaque buildup and helping stabilize existing plaques. While statins may not drastically shrink plaques, they improve their composition, making plaques less likely to rupture – a fact backed by imaging and histological evidence^[61,62]^. Taken together, these effects show why statins are so valuable for PAD patients, addressing both lipid and inflammatory pathways to protect cardiovascular and limb health.

### Antithrombotics

Antithrombotic therapy is key in treating PAD because it helps lower the risk of serious cardiovascular problems and limb complications like amputations and ischemia^[63,64]^. Beyond stopping clots from forming, these drugs also reduce inflammation, which adds to their effectiveness. Studies show that using low-dose rivaroxaban together with aspirin after procedures to reopen blocked vessels can significantly cut down major cardiovascular and limb events, though this does increase bleeding risk^[63,65]^. Large trials like COMPASS and VOYAGER-PAD found that blocking both clotting pathways works better than aspirin alone, especially for patients less likely to bleed^[63,65]^. Rivaroxaban works by blocking Factor Xa, which not only prevents clot formation but also reduces harmful immune cell buildup and inflammatory proteins inside plaques^[66]^. It also interferes with signals that trigger blood vessel lining activation, white blood cell sticking, and oxidative stress – all of which contribute to artery damage and unstable plaques^[66–68]^.

For patients who are more prone to bleeding, single antiplatelet drugs like clopidogrel are usually preferred^[63]^. Clopidogrel blocks the P2Y_12_ receptor on platelets, reducing factors that encourage platelets and white blood cells to stick together, which helps calm inflammation^[66]^. Other options, such as ticagrelor or aspirin combined with ticagrelor, also protect against major cardiovascular problems without causing as much bleeding as the dual pathway treatments^[69]^. Natural anticoagulants like antithrombin add another layer of protection by inhibiting clotting enzymes and lowering inflammation through reduced interactions between white blood cells and vessel walls^[68,70]^. Some traditional medicines, including nattokinase and Tongji 2 granules, have shown promise in blocking inflammatory pathways like IL-17A/nuclear factor kappa-light-chain-enhancer of activated B cells (NF-κB) and TLR4/NOX2/ROS/MAPK, cutting down cytokine release, immune cell buildup, and oxidative stress^[71,72]^. These help slow down the inflammatory environment caused by plaque damage and clot formation, preserving vessel function and slowing disease progression.

In summary, antithrombotic drugs do more than just prevent clots in PAD; they also help control inflammation, oxidative stress, and damage to the vessel lining – factors that worsen outcomes for patients^[69,73]^. This added anti-inflammatory effect, mainly by dampening cytokine signaling and activation of blood vessel cells, is an important bonus beyond their role in clot prevention^[66]^. Since PAD patients differ widely in their bleeding risks and disease severity, careful selection and ongoing monitoring of antithrombotic treatments are essential to get the best balance between reducing cardiovascular and limb risks and avoiding side effects^[69]^.

### Glycemic and Risk Factor Control

Effectively managing CLTI in people with diabetes depends heavily on controlling systemic inflammation by addressing key modifiable risk factors. These include maintaining stable blood glucose, quitting smoking, and managing blood pressure. Long-term high blood sugar contributes to blood vessel inflammation by encouraging the formation of advanced glycation end-products, which bind to RAGE receptors and activate harmful oxidative and inflammatory pathways, including NF-κB signaling^[74]^. This inflammatory environment plays a direct role in worsening PAD and accelerating its progression to CLTI. Clinical data show that every 1% increase in HbA1c correlates with a 28% increase in PAD risk among those with type 2 diabetes^[75]^. Short-term outcomes after revascularization are also worse in patients with poorly controlled diabetes. For example, HbA1c levels above 10% are strongly linked with higher rates of limb complications and amputation compared to levels in the 7–10% range^[76]^. In contrast, maintaining HbA1c below 7% brings complication rates down to levels similar to non-diabetics^[76,77]^.

Smoking remains another major contributor to inflammation and vascular damage. Chemicals in tobacco smoke induce oxidative stress and impair endothelial function, increasing the likelihood of plaque buildup and making blood vessels more prone to injury^[78]^. Continued smoking after a CLTI diagnosis worsens outcomes – speeding up disease progression, lowering graft survival, and raising the risk of limb loss. One study comparing persistent smokers to those who quit found that former smokers had significantly better 5-year outcomes, with amputation-free survival rates of 81 versus 60%, and lower overall mortality^[79]^. Blood pressure control plays a similarly important role. Elevated pressure damages vessel walls and promotes inflammation, particularly through angiotensin-II activity. Treatment with ACE inhibitors not only lowers blood pressure but also has an anti-inflammatory effect. One study found that ACEI users had over two times lower median CRP levels than patients on other blood pressure medications and experienced a 61% reduction in cardiovascular risk over 2 years^[80]^. This is likely due to their ability to reduce oxidative stress and inflammatory signaling by modulating the renin-angiotensin-aldosterone system (RAAS) pathway^[81]^.

In recent years, more targeted anti-inflammatory therapies have started to show promise alongside conventional risk factor control. Canakinumab, which blocks IL-1β, was shown in the CANTOS trial to significantly reduce major cardiac events – even without affecting cholesterol levels^[56,82]^. Similarly, the colchicine cardiovascular outcomes trial (COLCOT) trial demonstrated that colchicine, which modulates neutrophil activity, reduced ischemic events by 23%^[83]^. These findings suggest that inflammation-specific treatments may be valuable add-ons for patients with CLTI, especially when standard therapies are not enough. Combined with glycemic regulation, smoking cessation, and proper hypertension control, these therapies could offer a more complete strategy to prevent limb loss and lower cardiovascular risk. This layered, individualized approach is likely to be the most effective way forward for managing the complex and overlapping challenges posed by advanced PAD^[5]^.

## Investigational anti-inflammatory therapies

### Colchicine

Colchicine is a known anti-inflammatory agent traditionally used to treat gout and pericarditis^[84,85]^; however, its mechanism of action also makes it a promising option for targeting atherosclerotic inflammation^[86,87]^. Colchicine works by disrupting microtubule polymerization in leukocytes, neutrophil thereby impairing motility, adhesion, exocytosis, and mitosis^[88]^. In addition, colchicine also inhibits the NLRP3 inflammasome, thereby reducing the production of IL-1β and IL-18^[88]^ – two central cytokines implicated in the progression of atherosclerosis and vascular inflammation^[89]^. By stopping these inflammatory pathways, colchicine reduces endothelial injury, plaque disruption, and thrombosis, all of which are key mechanisms in both coronary artery disease (CAD) and PAD^[90]^.

Evidence from large cardiovascular trials has demonstrated colchicine’s capacity to reduce adverse cardiovascular events in patients with CAD (Nirdof *et al*, 2020)^[90]^. In the COLCOT trial, which enrolled 4745 patients who had recently experienced a myocardial infarction, 0.5 mg of Colchicine administered daily significantly reduced the risk of major adverse cardiovascular events (MACE) by 23% in comparison to placebo over a median follow-up of 22.6 months^[90]^. Similarly, the LoDoCo2 trial involving over 5500 patients with chronic coronary disease found a 31% relative reduction in the composite outcome of cardiovascular death, myocardial infarction, ischemic stroke, or urgent revascularization among colchicine users^[86]^. Subsequently several meta-analysis further confirm these findings and show that colchicine is associated with a statistically significant reduction in outcomes such as, cardiovascular death, myocardial infarction, and stroke in patients with atherosclerotic cardiovascular disease^[91,92]^.

Although patients with CLTI were not specifically studied in these trials, it is reasonable to consider that the benefits observed may also apply to them. This is because CLTI, which marks the most severe stage of PAD, shares the same underlying disease processes as CAD – namely, advanced atherosclerosis, persistent inflammation, and a tendency toward plaque instability and thrombosis^[93]^. Given that colchicine targets the very inflammatory pathways that contribute to plaque instability and thrombo-occlusion, its use in CLTI could potentially stabilize limb perfusion, reduce the need for repeat revascularization, and prevent tissue loss.

Several retrospective studies have investigated colchicine in patients with PAD, supporting this hypothesis^[93,94]^. A large retrospective analysis by Tramujas *et al* (2023)^[94]^ reported that colchicine use in patients with lower-extremity PAD was associated with a significantly reduced risk of major adverse cardiovascular and limb events, including a 16% reduction in amputations and 15% fewer revascularization procedures over 10 years. Furthermore, Lin *et al* (2024)^[93]^ study on over 60 000 patients with PAD showed that colchicine therapy was associated with a 25% reduction in MALE, along with decreased cardiovascular mortality. These findings suggest that the benefits observed in CAD may translate to PAD as well, particularly in high-risk subgroups such as those with CLTI. However, evidence from an emulated trial involving patients with gout and PAD presents a contrasting view. In this study, Heindel *et al* (2023)^[95]^ found no statistically significant difference in MALE or mortality at 2 years between colchicine users and those receiving non-steroidal anti-inflammatory drugs (NSAIDs). It is vital to interpret these results with caution as the study had several limitations. It was underpowered with a relatively small sample size and had a short follow-up period. Moreover, the non-randomized design leaves the findings susceptible to confounding factors that were not adequately controlled for. Notably, in a subset analysis of patients with CAD, the study also failed to replicate the positive outcomes reported in the COLCOT and LoDoCo2 trials, further questioning the reliability or generalizability of such findings. Given these conflicting findings and methodological limitations, only a well-designed, randomized controlled trial (RCT) focusing specifically on patients with CLTI can reliably determine the true efficacy of colchicine in this population.

### IL-1 inhibitors, canakinumab

Canakinumab is a human monoclonal antibody that exerts its anti-inflammatory effect by binding to IL-1β, thereby preventing its interaction with cell surface receptors^[96]^. IL-1β is predominantly activated by the NLRP3 inflammasome in response to cholesterol crystals, oxidative stress, or hypoxia – common features within atherosclerotic plaques^[97]^. This blockade interrupts downstream signaling pathways that drive systemic inflammation, resulting in reduced levels of circulating IL-6 and a decline in hepatic synthesis of acute-phase reactants such as CRP and fibrinogen – markers that have been linked to an elevated risk of cardiovascular events^[98,99]^.

The CANTOS trial provides evidence that targeting IL-1β in patients with CAD can lead to a reduction in cardiovascular events^[8]^. This multicenter, double-blind, RCT enrolled over 10 000 patients with a history of myocardial infarction and elevated high-sensitivity C-reactive protein (hsCRP ≥ 2 mg/L). Participants were assigned to receive subcutaneous injections of canakinumab at doses of 50, 150, or 300 mg, or placebo, administered every 3 months over a median follow-up of 3.7 years^[8]^. Only the 150 mg dose achieved a statistically significant reduction in the primary composite endpoint (non-fatal myocardial infarction, non-fatal stroke, or cardiovascular death) demonstrating a 15% relative risk reduction (hazard ratio 0.85, 95% confidence interval 0.74–0.98; *P* = 0.021)^[8]^. Results from patients who received the 50 and 300 mg doses did not meet the prespecified threshold for significance to draw any meaningful conclusions. However, canakinumab produced a significant, dose-dependent reduction in circulating levels of IL-6 and hsCRP level^[8]^, validating its anti-inflammatory mechanism of action.

Similarly, a double-blind, placebo-controlled trial by Russell *et al* (2019)^[100]^, where 38 patients with symptomatic PAD receiving monthly subcutaneous canakinumab 150 mg or placebo for 1 year, showed that levels of IL-6 and CRP decreased as early as 1 month following initiation of treatment, findings that are consistent with the anti-inflammatory effects previously demonstrated. However, despite these biochemical improvements, the study failed to achieve its primary endpoint as there were no significant change in plaque volume in either group on high-resolution 3.0 T magnetic resonance image scans of the superficial femoral artery, suggesting that canakinumab had no effect on plaque progression. Nevertheless, exploratory endpoints revealed potential functional benefits^[100]^. Patients receiving canakinumab experienced improvements in maximum walking distance and pain free walking distance, markers that reflect clinically meaningful gains in mobility and quality of life^[100]^. These outcomes while encouraging must be interpreted cautiously as the study was not specifically designed to evaluate exercise capacity. The study was also terminated early and recorded a significant loss of participants that may have affected overall results. Although canakinumab did not affect plaque regression in PAD, its anti-inflammatory effects and improvement in walking performance indicate potential benefits worth exploring in larger, targeted trials.

### Methotrexate

Methotrexate, a folate antagonist widely used in the treatment of inflammatory arthropathies, exerts complex immunomodulatory effects that extend beyond its anti-proliferative effects^[101]^. At low doses, methotrexate modulates cytokine expression, inhibits NF-κB activation, and promotes adenosine release, thereby causing anti-inflammatory effects without the cytotoxicity seen in oncological dosing^[102]^, which has led to investigation into its role in atherosclerosis.

In a study by Choi *et al* (2002)^[103]^, patients with rheumatoid arthritis treated with methotrexate exhibited a lower incidence of cardiovascular events, suggesting a possible protective effect. However, these findings were unfortunately not replicated when tested in a broader cardiovascular population^[104]^. The Cardiovascular Inflammation Reduction Trial (CIRT) enrolled patients with stable CAD and either type 2 diabetes or metabolic syndrome, randomizing them to low-dose methotrexate versus placebo. The trial failed to demonstrate any significant reduction in MACE, and methotrexate did not lower circulating levels of key inflammatory biomarkers, including IL-1β, IL-6, or hsCRP^[104]^.

A likely explanation for the failure of methotrexate in CIRT lies in its inability to effectively engage the IL-1β–IL-6–CRP inflammatory axis, a pathway that has been implicated in the pathogenesis of atherosclerosis^[105–107]^. In contrast, canakinumab, an IL-1β inhibitor tested in the CANTOS trial, demonstrated suppression of this pathway and was associated with reductions in MACE^[8]^. The difference in results shows that not all anti-inflammatory pathways are equal in the context of atherosclerosis. Agents that do not directly target the IL-1β–IL-6–CRP axis may have limited efficacy in this context^[108]^. Additionally, from these results, it may seem that baseline inflammatory status can influence therapeutic response. In CANTOS, the median baseline hsCRP was 4.2 mg/L, indicating a high inflammatory burden, while in CIRT, it was substantially lower at 1.6 mg/L. This difference could explain the lack of biomarker response and efficacy observed with methotrexate in CIRT. Although there is evidence showing that methotrexate is capable of reducing CRP levels, these data come mostly from studies conducted in acute inflammatory states, such as in active rheumatoid arthritis, where baseline CRP levels are markedly elevated^[105,109]^. Examining both trials, the results suggest that effective treatment of atherosclerotic inflammation likely depends on the precise targeting of the relevant immunopathogenic pathway and careful selection of patient populations with sufficient inflammatory activation to derive any benefit therapeutically.

### Emerging agents

#### NLRP3 inhibitors (e.g., MCC950) – preclinical

The NLRP3 inflammasome, part of the larger family of inflammasomes including nucleotide-binding and adaptive compounds with helical two-domain (NACHT), leucine-rich repeat (LRR), and pyrin domain (PYD) domain-containing proteins, plays a central role in how the body responds to infection and tissue damage. It is now understood to be involved in the regulation of several disease processes, including those seen in rheumatic, metabolic, and neurodegenerative conditions^[110]^. Like other inflammasome sensors, NLRP3 assembles into an innate immune complex in the cytosol. This leads to the activation of caspase-1, which then cleaves gasdermin D, setting off pyroptosis – a form of cell death that is tightly linked to inflammation. In particular, this process is a key mechanism of macrophage pyroptosis, which has been implicated in the progression of atherosclerosis^[111]^.

There is been growing interest in developing NLRP3 inhibitors to control inflammatory conditions^[112]^, but the challenge has been their broad effects. Because they interfere with multiple signaling pathways, these drugs can bring unwanted side effects. This has led researchers to shift focus toward designing more selective inhibitors that aim to target NLRP3 more precisely, reducing the risk of systemic impact^[112]^. In preclinical models, compounds like MCC950, CY-09, and OLT1177 have shown encouraging results – not only in terms of dampening inflammation but also in protecting tissue from damage^[113]^. Even so, moving these findings toward clinical application is not straightforward. Off-target effects remain a concern, and there is also the issue of potentially suppressing immune responses too much. Additionally, there is still uncertainty in identifying which patients would benefit most, and there is a risk that other inflammasomes, such as AIM2 or NLRC4, might compensate when NLRP3 is inhibited.

Zeng *et al* (2021)^[111]^ explored the effect of MCC950 and found that it led to smaller plaque areas and reduced macrophage content, pointing to a possible benefit in lowering the risk of PAD – something closely tied to CLTI. However, the study also showed that serum lipid levels in the aortic root showed no improvement, suggesting the drug’s effects may be limited to inflammation and cell death pathways, rather than lipid metabolism. Overall, the limited amount of solid preclinical data for some of these inhibitors, combined with the fact that others are still in early-stage clinical trials, means that their use in real-world inflammatory disease treatment remains somewhat out of reach for now.

#### IL-6 inhibitors (e.g., tocilizumab) – theoretical applications

IL-6 is a cytokine that plays several roles in the immune system, depending on the context. In healthy people, it is present in only tiny amounts, but its levels can spike dramatically – although briefly – in many kinds of pathological states^[114]^. Because of this pattern, IL-6 has become a major focus for treatments aimed at controlling inflammation, especially in diseases where the immune system is overactive. A lot of work has gone into understanding what IL-6 does and how it contributes to conditions like autoimmune diseases, some cancers, and chronic inflammation^[115]^.

Several drugs have been developed that target either IL-6 itself or its receptor. Among them, tocilizumab stands out – it is a monoclonal antibody that blocks the IL-6 receptor and has shown strong effectiveness in diseases like rheumatoid arthritis. It is currently the only IL-6-targeted therapy that has been approved for clinical use^[115]^. Another drug, siltuximab, which directly targets IL-6, has shown encouraging results in cancer settings, sometimes used on its own and sometimes alongside chemotherapy^[115]^. Beyond these, more IL-6-related therapies are still in the pipeline, with clinical trials ongoing across a range of diseases. While the theory behind targeting IL-6 is strong, many of these therapies are still being evaluated, and their place in routine clinical care is not fully defined yet.

### Nutraceuticals and experimental therapies

Omega-3 fatty acids play an important role in dampening inflammation by targeting several key steps in the inflammatory process. The two main types found in these fats – eicosapentaenoic acid (EPA) and docosahexaenoic acid (DHA) – have been shown to reduce the expression of adhesion molecules, lower leukocyte movement and attachment to blood vessel walls, and decrease the production of pro-inflammatory substances like prostaglandins and leukotrienes derived from arachidonic acid, an n-6 fatty acid^[116]^. These fatty acids also help to limit the release of inflammatory cytokines, which contribute to vascular inflammation.

EPA, in particular, generates specialized molecules such as resolvins, protectins, and maresins that actively resolve inflammation rather than just suppressing it. Research suggests that these effects come from changes to cell membrane fatty acid composition, disruption of lipid rafts, and inhibition of the pro-inflammatory transcription factor NF-κB^[116]^. Alongside omega-3s, compounds like resveratrol – a non-flavonoid polyphenol – have been suggested to reduce inflammation and support endothelial health, potentially slowing the progression of atherosclerosis, which is a major contributor to PAD and CLTI^[117]^.

Flavonoids, another group of polyphenols, are known for their antioxidant, anti-inflammatory, and various protective properties. Studies indicate that these hydroxylated compounds activate antioxidant pathways, inhibit the secretion of enzymes involved in inflammation like lysozymes and β-glucuronidase, and reduce arachidonic acid release, all of which help lower inflammatory responses^[118]^. Additionally, oxidative stress is a major obstacle to wound healing since excessive ROS damage cells and delay recovery. Small studies have shown that antioxidants can help by reducing this oxidative stress, thereby minimizing tissue damage and promoting better wound healing outcomes^[119]^. Table 1 below gives a summary of the various anti-inlfammatory agents that could have a role in managing CLTI.

**Table 1:** Anti-inflammatory agents

Agent	Mechanism of action	Key trial data	Relevance to CLTI
Colchicine	Inhibits microtubule polymerization; blocks NLRP3 inflammasome → ↓ IL-1β and IL-18	COLCOT: ↓ MACE by 23%^[90]^; LoDoCo2: ↓ CV events by 31%^[86]^; Meta-analyses support ↓ CV death and MI^[91]^	Retrospective PAD studies: ↓ amputations & revascularizations^[93,94]^; potential benefit, but no RCT in CLTI
Canakinumab	Monoclonal antibody targeting IL-1β → ↓ IL-6, CRP	CANTOS: ↓ MACE by 15% at 150 mg dose^[8,100]^: ↓ IL-6 and CRP in PAD but no plaque volume change	Suggests anti-inflammatory effect may improve walking distance; no plaque regression observed; limited PAD/CLTI evidence
Methotrexate	Anti-proliferative; promotes adenosine release → inhibits NF-κB and inflammatory cytokines	CIRT: No reduction in MACE or inflammatory markers^[104]^; Protective effect seen in RA cohorts only^[103]^	Ineffective in general atherosclerotic populations; may require high baseline inflammation; unlikely benefit in CLTI
NLRP3 inhibitors	Block activation of NLRP3 inflammasome → ↓ IL-1β, caspase-1, gasdermin D (↓ pyroptosis)	MCC950: ↓ plaque size and macrophages in mice^[111]^; no change in lipid levels; promising preclinical data	Theoretically promising for CLTI, but no clinical data yet; off-target effects and immune suppression remain concerns
IL-6 inhibitors	Block IL-6 or its receptor → ↓ CRP, systemic inflammation	Tocilizumab effective in RA; no major cardiovascular RCTs completed	Mechanistically attractive; data lacking in atherosclerosis and CLTI
Nutraceuticals	Omega-3s (↓ cytokines, adhesion molecules, produce resolvins); Polyphenols (↓ ROS, modulate inflammatory enzymes)	EPA: Anti-inflammatory effects via resolvins; Flavonoids: antioxidant and anti-inflammatory benefits; no RCT-level cardiovascular data	May aid wound healing and vascular function; supportive in adjunct role only; not suitable as monotherapy for high-risk CLTI patients

CIRT, Cardiovascular Inflammation Reduction Trial; CLTI, chronic limb-threatening ischemia; CRP, C-reactive protein; EPA, eicosapentaenoic acid; MACE, major adverse cardiovascular events; CV, cardiovascular death; MI, myocardial infarction; NF-κB, nuclear factor kappa-light-chain-enhancer of activated B cells; RA. rheumatoid arthritis, PAD, peripheral artery disease; RCT, randomized controlled trial; ROS, reactive oxygen species.

## Clinical considerations and challenges

### Limited RCTs and standardized care in CLTI

CLTI remains an area with relatively few RCTs. This scarcity stems from multiple challenges, including the complexity of the disease itself and difficulties in recruiting suitable volunteers. Ethical concerns around enrolling very ill patients also contribute. The varied nature of CLTI patients – who often suffer from multiple coexisting conditions like diabetes, heart disease, and kidney problems – makes it tough to isolate the effects of any one treatment. Moreover, differences in the location and severity of arterial blockages add further complexity for both clinicians and researchers.

The advanced stage of CLTI often comes with a high burden of comorbidities and shorter life expectancy, which leads many clinical trials to exclude these patients. Long-term follow-up is also difficult because many patients may drop out or pass away during the study, complicating the interpretation of results. Given these hurdles, focusing on patient-centered outcomes, such as quality of life or functional improvements, might be more appropriate for trials involving CLTI^[120]^. Another obstacle is the relatively small size of the CLTI patient population compared to earlier stages of PAD. The broad variation among these patients – in terms of age, race, sex, severity of disease, and anatomical differences – further complicates patient selection.

There is also the challenge of standardizing treatment and research protocols globally. Experience and resources vary widely between regions and medical centers^[121]^. Conducting trials that include a diverse, international patient and operator community would be a huge undertaking – perhaps an ideal but difficult goal. Still, recognizing these limitations is essential for designing better studies in the future.

### Balancing anti-inflammatory benefits and risks

Anti-inflammatory therapies come with their own risks, especially for patients with ulcerated or infected limbs. For example, corticosteroids have been linked to increased infection risks in patients with chronic limb ischemia, and some NSAIDs may worsen certain bacterial or viral infections, like those caused by Group A Streptococcus^[122]^. While these drugs can reduce symptoms related to inflammation, they may also delay infection diagnosis or increase susceptibility to infections. Choosing when and how to use these agents requires careful consideration of the drug’s properties, the infection type, and the patient’s overall condition.

Some CLTI patients are on systemic immunosuppressants for other conditions, raising questions about how these drugs affect limb outcomes. Research has found that long-term immunosuppression does not significantly impact late outcomes following first-time lower extremity revascularization, including vessel patency, reintervention rates, or major amputations^[123]^. However, other studies suggest that prolonged immunosuppressive therapy may raise infection risks in limb-threatening ischemia, which can lead to further complications and sometimes amputation. Deciding between anti-inflammatory agents and immunosuppressants depends heavily on the clinical context, existing comorbidities, and treatment goals^[124]^.

### Patient selection and biomarker use

Low-grade inflammation plays a role throughout the course of PAD – from its early development to progression and acute events like plaque rupture and thrombosis. Several inflammatory markers have been studied as potential indicators of disease severity and progression. Among these, CRP is the most commonly researched. Studies have found that CRP levels tend to be higher in PAD patients compared to controls and correlate with disease severity as measured by the ankle-brachial index^[125]^. CRP may be more useful for predicting short-term risks but is also linked to worse long-term outcomes, including major cardiovascular events and limb-related complications.

Because PAD is complex, relying on a single biomarker likely misses much of the disease’s underlying biology. Different inflammatory proteins can reflect distinct molecular pathways involved in vascular remodeling. For this reason, a multimarker approach might offer better insights into the patient’s condition. Other markers such as TNF-α, IL-6, fibrinogen, and D-dimer have also been helpful in assessing PAD severity and guiding treatment timing^[17,19]^.

### Polypharmacy and drug interactions in PAD patients

Taking multiple medications at once, or polypharmacy, is very common among people with PAD, especially those with other related vascular problems. One study looked at 354 patients and found that over half were taking five or more drugs, with nearly half of those taking 10 or more. Over nearly 5 years, those on a higher number of medications tended to have worse health outcomes, including a greater chance of major heart-related events. While we know that polypharmacy can have harmful effects in many illnesses, how exactly it affects patients with PAD is not fully understood yet. It is also unclear how different types of medications – whether cardiovascular drugs or others – impact risks and side effects^[126]^.

### Cost-effectiveness compared to limb salvage procedures

Inflammation is a key factor in the development of PAD, but most treatments do not directly tackle this issue. Using anti-inflammatory drugs to manage PAD is still not very common, even though it shows promise^[127]^. A common treatment for CLTI is angioplasty, which helps open blocked arteries. However, even after this procedure, death and amputation rates remain high, and many patients do not see much improvement in how well they can walk or function^[128]^.

The CANTOS study showed that lowering inflammation could reduce clot-related problems in heart disease, but the drug used, canakinumab, was very expensive – around $73 000 per year – and carried a risk of serious infections, so it was not widely adopted. Since then, researchers have turned their attention to more affordable anti-inflammatory medications like colchicine, which is already used for conditions like gout and pericarditis. Big trials like COLCOT and LoDoCo2 have looked at colchicine for heart disease prevention, and future studies will hopefully show whether it can help reduce the remaining heart and limb risks in PAD patients as well^[127]^.

## Future directions and research priorities

### Designing CLTI-specific clinical trials for inflammation-modulating drugs

Most inflammation-targeting drugs currently being tested focus on heart disease and atherosclerosis, but there is a clear need for studies specifically looking at these drugs in patients with PAD and CLTI. Right now, treatments for CLTI mainly revolve around managing infections, pain relief, and surgical or endovascular procedures to restore blood flow. Researchers are exploring new therapies like special exercise programs, novel drugs, stem cell treatments, and RNA-based therapies. Several clinical trials exploring these options are expected to report results within the next few years^[129]^.

Running large clinical trials requires a lot of infrastructure, which until recently has been built separately for each drug development project. Only a handful of big companies can afford to manage these resources on their own. To make this easier, many companies work with academic research organizations or contract research groups that handle parts of the trial process – like developing protocols, managing trial sites, or overseeing data and safety. Outsourcing lets companies convert some fixed costs into flexible ones and tap into specialized expertise that would be hard to develop internally^[130]^.

### Using inflammatory biomarkers to guide treatment

Inflammation plays a central role in PAD and CLTI, but exactly which molecules are key players is not fully clear. Many studies have looked at inflammatory markers involved in early atherosclerosis, including cytokines like IL-1β, IL-6, CRP, and others that promote immune cell attraction and vessel wall damage. However, these markers have not been widely studied specifically in CLTI patients.

Among these markers, CRP, fibrinogen, D-dimer, IL-6, IL-8, and the NLR have fairly strong evidence backing their use as indicators of disease severity or prognosis. Other markers – such as sortilin, OPG, HMGB-1, neopterin, pentraxin-3, calprotectin, and NGAL – still need more research to confirm their role in PAD and whether they could help predict outcomes or guide treatment decisions. Researchers are also paying attention to extracellular vesicles and micro-RNAs, which might prove important in understanding and monitoring CLTI^[19]^.

### Combining anti-inflammatory treatments with revascularization and wound care

Some studies suggest that short-term, low-dose use of NSAIDs and COX-2 inhibitors does not significantly harm soft tissue healing, though these drugs might slow bone healing. Still, more carefully controlled human studies are needed to understand how these medications affect wound healing in CLTI, especially when combined with revascularization procedures^[131]^.

There is evidence that managing inflammation promptly can speed up recovery from CLTI by up to 3 months. Measuring inflammatory markers during and after healing supports the idea that targeted anti-inflammatory therapies could improve outcomes. For example, canakinumab, an IL-1β blocker, has been shown to improve walking ability in patients with claudication while lowering CRP and IL-6 levels. Statins also reduce systemic inflammation and have been linked to better survival, fewer major cardiovascular events, and longer periods without amputation in CLTI patients, largely due to their anti-inflammatory effects^[22]^.

### The role of artificial intelligence in predicting inflammation-related outcomes

Artificial intelligence (AI) is opening new possibilities in medical diagnosis and treatment by processing large amounts of complex data far faster than humans can. AI can spot subtle patterns that might be missed otherwise, offering a more detailed and objective analysis of patient information^[132]^.

AI covers a wide range of techniques, including natural language processing, machine learning, deep learning, computer vision, and more^[133]^. Researchers are exploring how AI and machine learning can help address health disparities in CLTI patients by improving diagnosis, guiding treatment, monitoring progress, and predicting outcomes. Early AI applications in CLTI are growing quickly, offering hope for better care – especially in underserved communities^[134]^.

## Conclusion

CLTI is a severe manifestation of PAD, driven by both atherosclerotic occlusion and chronic inflammation. Targeting inflammation has emerged as a promising adjunct to conventional therapies like revascularization and antiplatelet treatment. Among available agents, colchicine has demonstrated consistent cardiovascular benefits and potential limb-related advantages, although data in CLTI-specific populations remain limited. Canakinumab, an IL-1β inhibitor, has shown anti-inflammatory and functional benefits in early PAD studies, while methotrexate failed to improve outcomes, highlighting the importance of targeting the right inflammatory pathways.

Emerging therapies, such as NLRP3 inflammasome inhibitors, offer mechanistic promise but are still in preclinical stages. Nutraceuticals like omega-3 fatty acids and polyphenols may provide mild adjunctive benefits but lack robust clinical evidence in CLTI.

Ultimately, while inflammation-modulating therapies show potential to enhance CLTI management, their clinical utility remains unproven without dedicated randomized trials. Future research should focus on high-risk subgroups, biomarker-driven patient selection, and integrated strategies that combine immunomodulation with established vascular interventions.

## Data Availability

Data sharing is not applicable to this article as no datasets were used.
